# Artificial intelligence-based prediction of overall survival in metastatic renal cell carcinoma

**DOI:** 10.3389/fonc.2023.1021684

**Published:** 2023-02-16

**Authors:** Ella Barkan, Camillo Porta, Simona Rabinovici-Cohen, Valentina Tibollo, Silvana Quaglini, Mimma Rizzo

**Affiliations:** ^1^ Department of Artificial Intelligence for Accelerated Healthcare & Life Sciences Discovery, IBM Research - Israel, University of Haifa Campus, Haifa, Israel; ^2^ Department of Interdisciplinary Medicine, School of Medicine, University of Bari Aldo Moro, Bari, Italy; ^3^ Division of Medical Oncology, Azienda Ospedaliero Universitaria Consorziale Policlinico di Bari, Bari, Italy; ^4^ Laboratory of Informatics and Systems Engineering for Clinical Research, Scientific Clinical Institute Maugeri (ICS Maugeri), Pavia, Italy; ^5^ Department of Electrical, Computer and Biomedical Engineering, University of Pavia, Pavia, Italy

**Keywords:** artificial intelligence, machine learning, predictive model, overall survival, metastatic renal cell carcinoma, first-line treatment

## Abstract

**Background and objectives:**

Investigations of the prognosis are vital for better patient management and decision-making in patients with advanced metastatic renal cell carcinoma (mRCC). The purpose of this study is to evaluate the capacity of emerging Artificial Intelligence (AI) technologies to predict three- and five-year overall survival (OS) for mRCC patients starting their first-line of systemic treatment.

**Patients and methods:**

The retrospective study included 322 Italian patients with mRCC who underwent systemic treatment between 2004 and 2019. Statistical analysis included the univariate and multivariate Cox proportional-hazard model and the Kaplan-Meier analysis for the prognostic factors’ investigation. The patients were split into a training cohort to establish the predictive models and a hold-out cohort to validate the results. The models were evaluated by the area under the receiver operating characteristic curve (AUC), sensitivity, and specificity. We assessed the clinical benefit of the models using decision curve analysis (DCA). Then, the proposed AI models were compared with well-known pre-existing prognostic systems

**Results:**

The median age of patients in the study was 56.7 years at RCC diagnosis and 78% of participants were male. The median survival time from the start of systemic treatment was 29.2 months; 95% of the patients died during the follow-up that finished by the end of 2019. The proposed predictive model, which was constructed as an ensemble of three individual predictive models, outperformed all well-known prognostic models to which it was compared. It also demonstrated better usability in supporting clinical decisions for 3- and 5-year OS. The model achieved (0.786 and 0.771) AUC and (0.675 and 0.558) specificity at sensitivity 0.90 for 3 and 5 years, respectively. We also applied explainability methods to identify the important clinical features that were found to be partially matched with the prognostic factors identified in the Kaplan-Meier and Cox analyses.

**Conclusions:**

Our AI models provide best predictive accuracy and clinical net benefits over well-known prognostic models. As a result, they can potentially be used in clinical practice for providing better management for mRCC patients starting their first-line of systemic treatment. Larger studies would be needed to validate the developed model

## 1 Introduction

For metastatic renal cell carcinoma (mRCC), two main prognostic systems have been developed and validated over the years. The first of these systems was developed by Robert J. Motzer and his colleagues, who assessed 670 patients enrolled in clinical trials of cytokine-based immunotherapy (or chemotherapy) at the Memorial Sloan Kettering Cancer Center (MSKCC) (1) and further validated in (2). Multivariate analysis showed that hemoglobin, serum lactate dehydrogenase, corrected serum calcium level, nephrectomy status, and Karnofsky Performance Status (KPS) were independent risk factors for the prediction of survival. Using a combination of these individual factors, patients were stratified as being of good, intermediate, or poor risk, with mean survival times of 20, 10, and 4 months, respectively. As a whole, this score is better known as the MSKCC (or Motzer) score.

In 2009, the International Metastatic Renal Cell Carcinoma Database Consortium (IMDC) retrospectively reviewed 645 consecutive patients from 7 different centers, treated with molecularly targeted agents; they developed a novel prognostic model (3) known as the IMDC (or Heng) score. This model was subsequently externally validated (4) for patients treated with immune checkpoint inhibitors (5) and in different treatment lines (6, 7). According to the Heng score, independent predictors of short overall survival (OS) are hemoglobin levels below the lower limit of normal (LLN), corrected calcium values greater than the upper limit of normal (ULN), KPS <80%, time from diagnosis to treatment < 1 year, neutrophils > ULN, and platelets > ULN. Patients were grouped according to the number of prognostic factors into a good risk group (0 factors), an intermediate risk group (1–2 factors), and a poor risk group (3–6 factors). For the good, intermediate, and poor risk groups, the median OS and 2-year OS were the following: not reached, 27 months, and 8.8 months; and 75%, 53%, and 7%, respectively (3).

With the continuing evolution of available technologies, studies using Artificial Intelligence (AI) have also been introduced. Recent studies for predicting long-term outcomes like survival and recurrence include the following works: Byun et al. (8) applied deep learning for prediction of prognosis of nonmetastatic clear cell renal cell carcinoma, Kim et al. (9) applied machine learning (ML) for predicting the probability of recurrence of renal cell carcinoma in five-years after surgery Guo et al. (10) compared a neural network with a boosted decision tree model to predict recurrence after curative treatment of RCC.

With the availability of multi-modal data sets that combine clinicopathological data with molecular, genetic, and imaging modalities, more studies for predicting long term outcomes have been carried out. Duarte et al. (11) compared different data mining techniques for predicting 5-year survival, based on a dataset obtained from The Cancer Genome Atlas. Zhao et al. (12) proposed a prognostic model by combining clinical and genetic information that monitors the disease progression in a dynamically updated manner. It is important to mention that availability of multi-modal data sets is limited. In our study, we are going to develop prediction models using only clinicopathological data. According to our knowledge, no such studies have been conducted to predict the survival of mRCC patients starting systemic treatment for clinicopathological data using AI.

The objective of the current study is to deploy an innovative machine learning method to develop novel predictive models for mRCC patients using Real World Data, in terms of three- and five-year survival rates from the time they started first-line systemic treatment, and compare these methods with available prognostic models.

## 2 Materials and methods

### 2.1 Patients

Data were collected from 342 consecutive mRCC patients who started first-line systemic therapy (with or without combined locoregional treatments, e.g., radiotherapy) between March 1, 2004, and March 1, 2019. The last follow-up was December 31, 2019.

The data were retrospectively collected from the patients’ hospital records and organized in an electronic Case Report Form (eCRF), using the REDCap platform. For each patient, the following information was retrieved: demographics, clinical characteristics, tumor characteristics, cancer history (including all systemic treatment lines), number and sites of metastases, as well as severe treatment-related adverse events (AEs), possibly affecting treatment delivery. Laboratory examinations were available at the beginning of each treatment line. In addition, time intervals and episodes derived from the oncological history were available (e.g., date of RCC diagnosis, surgery, diagnosis of metastatic disease, and start and end of each treatment line). Response to treatment was evaluated by the two treating physicians, no central review having been performed given the nature of this case series.

The data used for analysis was pseudonymized by removing all personal information and adding random noise to dates and numerical values, while maintaining the same clinical meaning of the original data.

**Figure 1:**
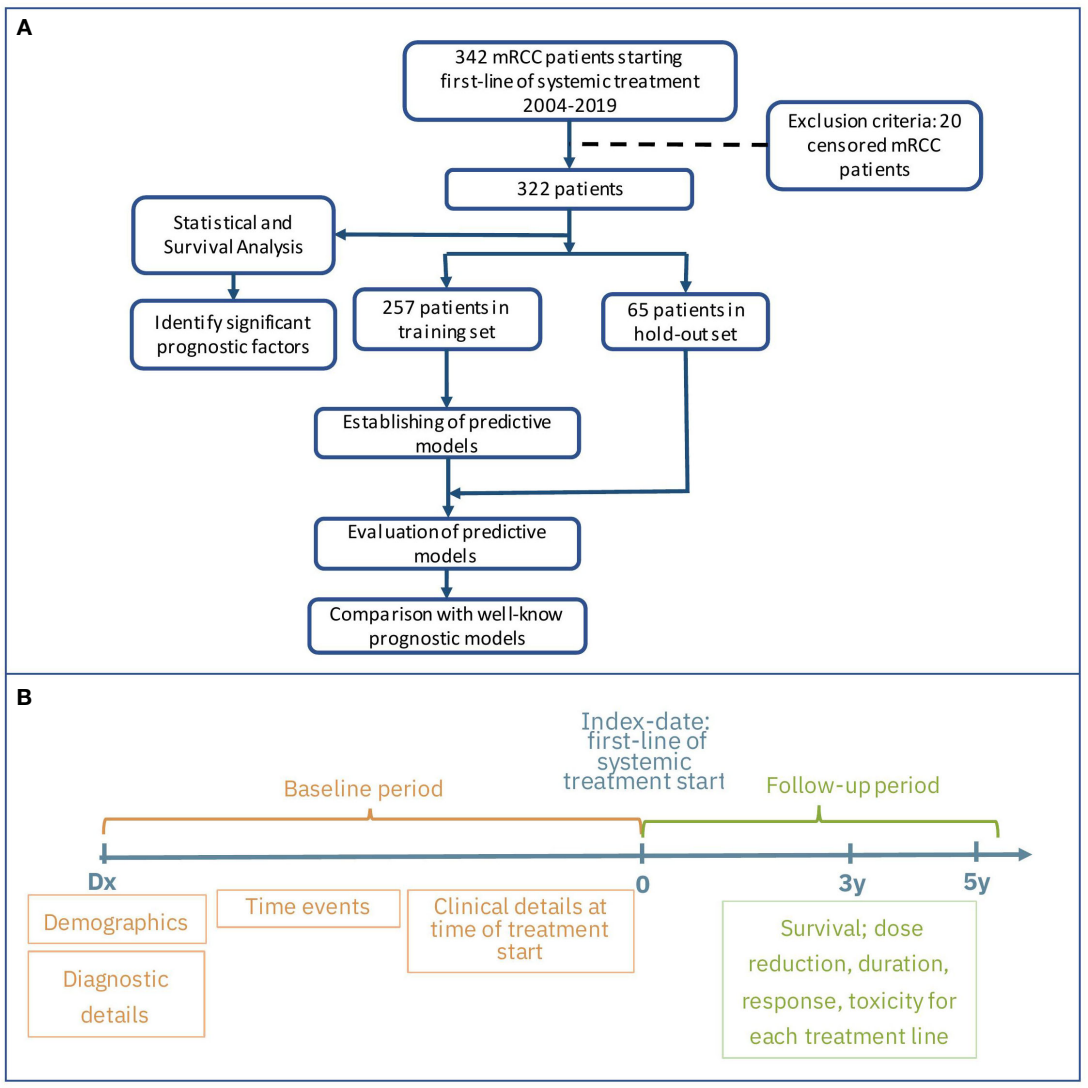
Flow chart of study design **(A)** and main groups of features and outcomes **(B)**.

We excluded 20 patients who were alive at the follow-up cut-off date, but had a follow-up time of less than 5 years. The remaining 322 patients were included in our study and were used for statistical analysis, model development, and testing (**Figure 1A**).

This study was approved by the Ethics Committee of IRCCS Istituti Clinici Scientifici Maugeri (Approval Number 2421 CE) on February 23, 2020.

### 2.2 Statistical analysis

Statistical analysis was applied to all patients in our study. For continuous variables, the data were presented as median and interquartile range (IQR), and for categorical variables, as frequencies (%). We conducted a Wilcoxon rank sum or Chi-square-square test and Fisher’s test to compare continuous or categorical variables, respectively. A p-value of <0.01 was considered statistically significant. These statistical tests were applied to compare baseline characteristics of different patient groups.

We used the univariate Cox proportional hazards model (13) to compare the correlation between different clinical characteristics and the overall survival from the first treatment line. Kaplan-Meier analysis (14) with the log-rank test was also performed for each variable. Significant covariates (p-value < 0.01) from the univariate Cox and Kaplan Meier analyses were included in the multivariate Cox regression model. During the building of the model, we excluded variables with a p-value > 0.1 under verification, with no significant changes in the multivariate estimators of HR. A p-value < 0.01 was considered significant for this model. We also analyzed the association of several intermediate outcomes (number of treatment lines; toxicity, dose reduction and response in the first treatment line) with survival by checking statistical significance in the univariate analyses. We further adjusted the developed multivariate Cox model to one of them (number of treatment lines) by incorporating this covariate into the model in a time-dependent manner. We used the R 4.1.3 gtsummary (15) and survival (16) packages and the Python (version 3.9.0) pandas library (17) for data description and for all statistical analyses.

### 2.3 Feature selection and data preprocessing

The aim of this research is to develop a new machine learning model that can predict OS for mRCC patients starting first-line systemic treatment, based on their clinico-pathological factors. **Figures 1A, B** presents the study design and main groups of patient details for the baseline and follow-up periods. For model development, we used patient details collected at the baseline period before treatment started. Because our data were derived from real-world settings, we are missing values for about 5% of the patients who didn’t undergo surgery, and are missing histopathological and staging details. We applied several methods to resolve any missing data, and this allowed us to include all patient details in the model development. One method was based on inference from other variables approved by medical experts. For example, for the M stage, if the time interval between the RCC and mRCC diagnoses was less than two months, it was considered as the presence of metastases at diagnosis time and the M1 value was imputed; accordingly, the M0 value was imputed in the opposite case. The mean imputing was applied for the rest of the variables. Standardization of all variables was done by removing the mean and scaling to unit variance.

We used the feature extraction tool described in Ozery-Flato et al. (18) to extract all the features and outcomes of the cohort’s patients. The process included several steps. In the first step, we included all demographic, histopathological, and clinical variables readily available from before treatment. More details can be seen in **Figure 1B**. Next, using date information variables, we generated new features that indicate durations between important events, e.g., time from diagnosis to surgery, time from diagnosis to first systemic treatment, etc. Following that, guided by well-known clinical knowledge approved by medical experts, we enriched the feature set by transformations and extensions of the variables. This included type transformation, such as converting categorical features to binary ones by grouping their values and converting continuous variables to binary by splitting them into groups using predefined thresholds. For example, the categorical N stage was converted to a binary value by grouping the N1 and N2 stages into one class, and the rest into another. In a similar fashion, the Fuhrman grade was transformed by grouping grade I or II vs. grade III or IV. For continuous variables of lab tests, we generated indicators of elevating predefined thresholds using clinically approved exam limits (e.g., low hemoglobin). Following recent research publications (19, 20), beyond the raw exam values, we also considered promising prognostic predictors, namely NLR (Neutrophils Lymphocytes Relation) and PLR (Platelets Lymphocytes Relation). We managed both the original and the transformed variables in the features set, allowing the predictive model to prioritize the feature importance and obtain better results.

Moreover, we enriched our feature set with nine new variables that represent patients’ risk scores calculated according to the well-known prognostic models of OS prediction for mRCC. The wide set of clinicopathological characteristics available in our data set for both diagnosis and treatment start time points allowed us to generate patient risk scores for the long list of prognostic models. We added five features of risk scores using the following models that predict OS and progression-free survival (PFS) from the time of RCC diagnosis: AJCC TNM staging system (21); SEER staging system (22); UCLA Integrated Staging System (UICC) (23); Stage, Size, Grade, and Necrosis model (SSIGN) (24); and Leibovich model for progression prediction (25). In addition, we added four features of risk scores using the following models predicting OS from the first-line of systemic treatment for mRCC patients: MSKCC (1); IMDC (3); International Kidney Cancer Working Group (IKCWG) model (26); and Modified International Metastatic Renal Cell Carcinoma Database Consortium (Modified IMDC) (19).

### 2.4 Development of predictive models

Prior to the development of predictive models, the cohort was split into two mutually exclusive sets. We assigned 80% of the patients to a training set, which was used for model generation. The remaining 20% of the data was defined as a hold-out. To ensure a good balance of patients while splitting a small data set, we used stratification on the outcomes and the top significant features from the univariate analysis. We then verified the feature distribution between the training and hold-out sets using statistical analysis methods (see **Tables 1A**, **B**).

**Table 1A:** The clinicopathological characteristics of the RCC patients from this study avaialble at the diagnosis time.

Characteristic	Total, N = 322^1^	Train, N = 257^1^	Hold-Out, N = 65^1^	p-value^2^
Gender				0.96
Male	252 (78%)	201 (78%)	51 (78%)	
Female	70 (22%)	56 (22%)	14 (22%)	
Age at diagnosis				0.56
< 65	254 (79%)	201 (78%)	53 (82%)	
>= 65	68 (21%)	56 (22%)	12 (18%)	
T stage				0.26
T1	45 (15%)	35 (14%)	10 (17%)	
T2	66 (21%)	49 (20%)	17 (28%)	
T3	189 (62%)	158 (64%)	31 (52%)	
T4	7 (2.3%)	5 (2.0%)	2 (3.3%)	
M stage				0.15
M0	202 (63%)	167 (65%)	35 (54%)	
M1	61 (19%)	48 (19%)	13 (20%)	
MX	59 (18%)	42 (16%)	17 (26%)	
N stage				0.90
N0	168 (52%)	132 (51%)	36 (55%)	
N1	37 (11%)	30 (12%)	7 (11%)	
N2	13 (4.0%)	10 (3.9%)	3 (4.6%)	
NX	104 (32%)	85 (33%)	19 (29%)	
Tumor Size				0.72
0-40 mm	15 (4.8%)	12 (4.8%)	3 (4.8%)	
40-70 mm	94 (30%)	78 (31%)	16 (25%)	
70-100 mm	126 (40%)	97 (38%)	29 (46%)	
> 100 mm	80 (25%)	65 (26%)	15 (24%)	
Fuhrman Grade				0.41
Grade I	5 (1.6%)	3 (1.2%)	2 (3.3%)	
Grade II	103 (34%)	81 (33%)	22 (37%)	
Grade III	121 (39%)	97 (39%)	24 (40%)	
Grade IV	78 (25%)	66 (27%)	12 (20%)	
Microvascular Invasion	128 (42%)	103 (42%)	25 (42%)	>0.99
Intra-Tumoral Necrosis	194 (63%)	158 (64%)	36 (60%)	0.57
Histology Groups				0.33
Clear Cell RCC	289 (90%)	231 (90%)	58 (89%)	
Papillary RCC	25 (7.8%)	20 (7.8%)	5 (7.7%)	
Chromophobe RCC	3 (0.9%)	1 (0.4%)	2 (3.1%)	
Unclassified RCC	3 (0.9%)	3 (1.2%)	0 (0%)	
Collecting Duct Carcinoma	2 (0.6%)	2 (0.8%)	0 (0%)	
Sarcomotoid Feature	83 (26%)	70 (27%)	13 (20%)	0.23

^1^n (%); Median (IQR).

^2^Pearson’s Chi-squared test; Fisher’s exact test; Wilcoxon rank sum test.

Missing diagnostic details for 5% of patients that didn’t undergo nephrectomy are not included.

**Table 1B:** The clinicopathological characteristics avaialable at start of the first systemic treatment line and the outcomes for the RCC patients from this study.

Characteristic	Total, N = 322^1^	Train, N = 257^1^	Hold-Out, N = 65^1^	p-value^2^
BMI group				0.94
Normal	136 (42%)	109 (42%)	27 (42%)	
Overweight	155 (48%)	124 (48%)	31 (48%)	
Obese	31 (9.6%)	24 (9.3%)	7 (11%)	
Karnofsky PS				0.38
80	17 (5.3%)	13 (5.1%)	4 (6.2%)	
90	57 (18%)	42 (16%)	15 (23%)	
100	248 (77%)	202 (79%)	46 (71%)	
Kidney Metastases	56 (17%)	46 (18%)	10 (15%)	0.63
Lymphnodes Metastases	145 (45%)	119 (46%)	26 (40%)	0.36
Lung Metastases	235 (73%)	184 (72%)	51 (78%)	0.27
Brain Metastases	15 (4.7%)	11 (4.3%)	4 (6.2%)	0.51
Liver Metastases	59 (18%)	50 (19%)	9 (14%)	0.30
Bone Metastases	69 (21%)	51 (20%)	18 (28%)	0.17
Hemoglobin g/dl	13.8 (12.7, 14.7)	13.8 (12.7, 14.6)	13.9 (12.5, 15.1)	0.69
Serum Corr. Calcium mg/dl	9.8 (9.3, 10.1)	9.8 (9.3, 10.1)	9.8 (9.5, 10.1)	0.68
LDH mU/ml	233.0 (199.2, 315.0)	232.0 (200.0, 310.0)	235.0 (199.0, 337.0)	0.47
Serum Sodium (Na) mmol/l	141.0 (139.0, 144.0)	141.0 (139.0, 144.0)	142.0 (140.0, 144.0)	0.11
Creatinine	1.1 (0.9, 1.3)	1.1 (0.9, 1.3)	1.2 (1.0, 1.3)	0.069
PLR	170.3 (123.7, 218.2)	170.6 (124.0, 215.0)	168.2 (122.2, 240.0)	0.53
NLR	3.5 (2.8, 4.2)	3.5 (2.9, 4.2)	3.5 (2.6, 4.5)	0.74
Months from Dx to 1st line	12.8 (3.7, 49.6)	12.4 (3.9, 47.1)	12.9 (3.6, 56.0)	0.92
Days from Dx to Surgery	19.0 (10.0, 32.0)	18.0 (10.0, 32.0)	20.5 (10.0, 37.0)	0.85
Treatment - 1st line				0.68
VEGF/VEGFRi* - mono	273 (85%)	217 (84%)	56 (86%)	
mTORi* - mono	10 (3.1%)	8 (3.1%)	2 (3.1%)	
IO – IO	4 (1.2%)	3 (1.2%)	1 (1.5%)	
IO - VEGF/VEGFRi*	20 (6.2%)	15 (5.8%)	5 (7.7%)	
Cytotoxic Chemotherapy	15 (4.7%)	14 (5.4%)	1 (1.5%)	
3y OS	125 (39%)	100 (39%)	25 (38%)	0.95
5y OS	68 (21%)	55 (21%)	13 (20%)	0.80
Number of Treatment Lines	3.0 (2.0, 3.0)	3.0 (2.0, 3.0)		0.85
Toxicity, grades 3-4 - 1st line	116 (36%)	90 (35%)	26 (40%)	0.45
Dose Reduction - 1st line	98 (30%)	75 (29%)	23 (35%)	0.33
Best Response - 1st line				0.18
Complete Response	10 (3.1%)	9 (3.5%)	1 (1.5%)	
Partial Response	117 (36%)	95 (37%)	22 (34%)	
Stable Disease	160 (50%)	130 (51%)	30 (46%)	
Progressive Disease	35 (11%)	23 (8.9%)	12 (18%)	

^1^n (%); Median (IQR).

^2^Pearson’s Chi-squared test; Fisher’s exact test; Wilcoxon rank sum test.

IO, Immuno-Oncology; i*, inhibitors.

**Figure 2:**
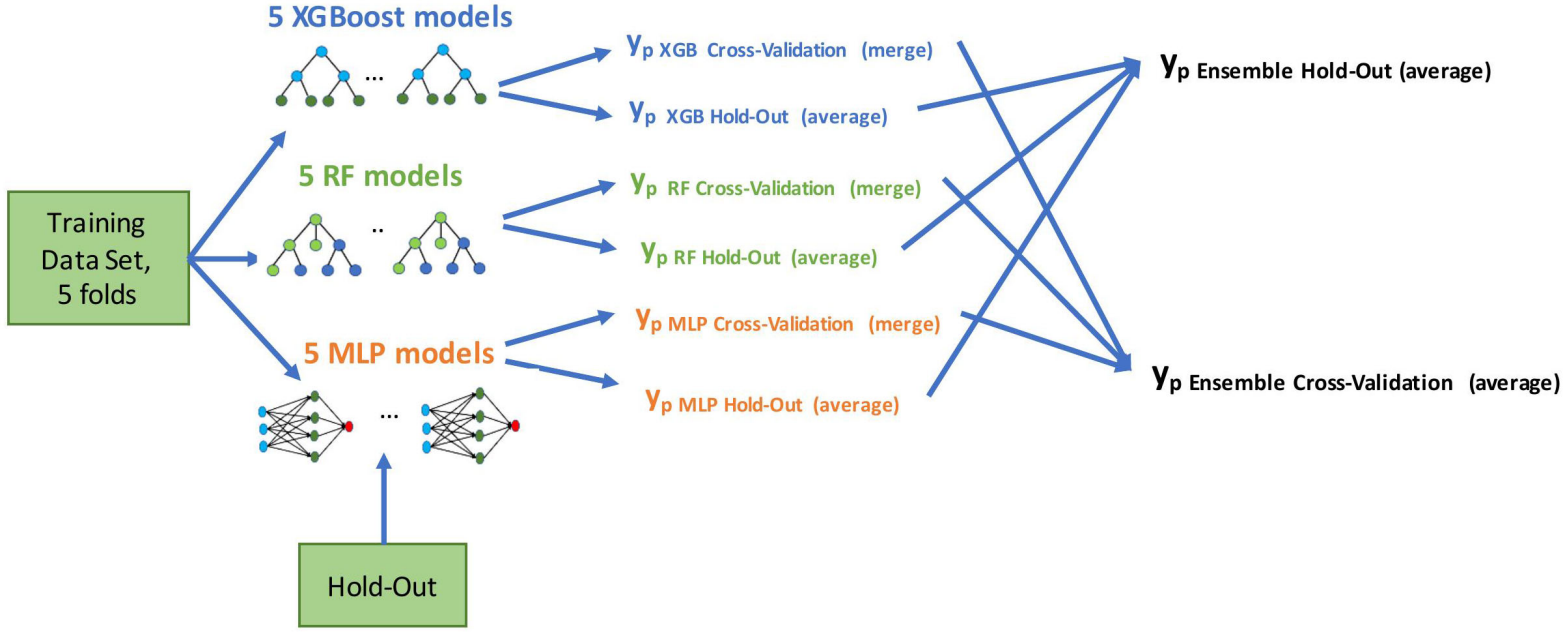
Model development flow. We built five models using training data in a cross-validation method (four folds for training, one fold for evaluation, five times in a round-robin manner). Evaluation was done on both cross-validation and hold-out predictions; y_p_ marks a vector of predicted probabilities.

We developed the models using a five-fold cross-validation method on the training set. We used all the features for model development, allowing automatic feature selection in the model. Multicollinearity was present in the baseline clinical data, (e.g., correlation between M stage and time from RCC diagnosis to first-line treatment start) and was further extended with risk scores of the well-known prognostic models. Therefore, we used state-of-the-art tree-based models and neural nets that work well with multicollinear data: eXtreme Gradient Boosting (XGBoost) (27), Random Forest (RF) (28), and Multilayer Perceptron (MLP) (29). We applied a grid-search method to determine the optimal parameters for predictive models. Initially, we built the models using these three selected methods. We then combined them in an ensemble by averaging the predictive probabilities of the individual models. Using the ensembling method allowed us to improve the generalization and reduce the variance of the different models, which leads to a more stable and robust final model. The final ensemble model was evaluated on cross-validation and hold-out cohorts. As depicted in **Figure 2**, we generated final prediction probabilities for the hold-out set as averages of the predictions from each of the five models. These prediction averages were further averaged in the ensemble model. For the cross-validation set, we merged the final probabilities of five cross-validation models into one vector before the ensembling stage.

We used the Python scikit-learn 0.24.2 package (30) to construct a predictive machine learning model. MLP can be developed with the open source FuseMedML (31), a PyTorch-based deep learning framework for medical data.

### 2.5 Evaluation of predictive models

We evaluated the developed models in a five-fold cross-validation on the training data set and on the hold-out data. The predictive performance of models was quantified using receiver operating characteristic (ROC) curve analysis, and its predictive accuracy was assessed by the area under the ROC curve (AUC) with 95% confidence intervals (32), and by assessing specificity at high sensitivity operation points. We used the DeLong test (33) to assess the significance between two AUCs computed on the same cohort and the McNemar test (34) to verify the significance between model results at high sensitivity points. The results of the latter test are important for models deployed in clinical practice, and thus are of special interest. In addition, we performed subgroup analysis for the ensemble model to detect sub-cohorts in which specific models demonstrated high quality results and were considered more reliable for these sub-populations during their usage in clinical practice. Also, we validated the results of the ensemble model for the sub-cohorts corresponding to low (1-2) and high (>=3) number of treatment lines that might be associated with survival. The FuseMedML (31) open source package was used to perform all the above-mentioned evaluation methods and tests.

We used Shapley additive explanations (SHAP) (35) to understand the machine learning predictive models and to explain feature importance; these demonstrated how each feature of each patient affects the predictive model results. We assessed the clinical usefulness and net benefits of the developed models using Decision Curve Analysis (DCA) (36, 37). DCA estimates the net benefit of a model by calculating the weighted difference between the true- and false-positive rates, where weighting between them is done with the odds of the threshold probability of the involved clinical risk. We compared the performance of the final ensemble model with the performance of four well-known models applied to our data set for survival prognosis. Specifically, we carried out comparisons with validated MSKCC (2), IKCWG (26), validated IMDC (4), and Modified IMDC (19).

## 3 Results

### 3.1 Baseline characteristics and survival analysis

The entire study cohort consisted of 322 patients. The median age of the study group at RCC diagnosis was 56.7 years (IQR 47.3 to 63.7 years) and 78% of participants were male. All patients were white. Clear cell type was diagnosed in 90% of patients and 95% of patients underwent cytoreductive nephrectomy. The median survival time from first-line of systemic treatment was 29.2 months (IQR 16.2 to 53.0 months) and 307 (95%) patients died during the follow-up period. The median PFS time was 8.1 months (IQR 0.5 to 37.3 months) and the median time from diagnosis to the first line of systemic treatment was 12.8 months (IQR 3.7 to 49.6 months). As part of a first-line systemic treatment, 85% of the patients received VEGFR/VEGFR inhibitor (VEGF/VEGFRi) monotherapy, 6.2% received a combination of immunotherapy (IO) and VEGF/VEGFRi (referred to here as the “IO-VEGF/VEGFRi combination”), 4.7% received cytotoxic chemotherapy, 3.1% received mTOR inhibitor (mTORi) monotherapy, and 1.2% received a combination of two immunotherapies (referred to here as the “IO-IO combination”). During the first-line treatment, 39.1% of the patients demonstrated an objective response, and 36% experienced adverse events of grade 3 and 4. The full summary of patient characteristics can be found in **Tables 1A**, **B** together with the verification of balancing between training and hold-out cohorts. **Supplementary Tables 1**, **2** summarize the results of the univariate analysis with respect to 3 and 5 year survival times, respectively. Significant baseline characteristics (with p<0.01) for both outcomes include the time from Dx to first-line treatment, age at diagnosis, Karnofsky PS, M stage, Fuhrman grade, sarcomatoid feature, LDH, and hemoglobin and PLR values. In addition, tumor size, microvascular invasion, lymph node and lung metastases were found to be significant for 3 year survival, and NLR was found to be significant for 5 year survival times.

As a result of the univariate survival analysis, several values of baseline clinico-pathological variables were defined as risk factors with p<0.01 in both methods: univariate Cox regression analysis and Kaplan–Meier log-rank tests. These variables include age at the time of the RCC diagnosis, M stage, time from Dx to first-line treatment, sarcomatoid feature, Karnofsky performance score, bone and brain metastases, hemoglobin, LDH, NLR, serum corrected calcium, and serum sodium (Na). In addition, the Fuhrman grade was found significant in Kaplan-Meier. Further details about the Cox regression analysis and Kaplan–Meier log-rank can be found in **Table 2**. The IO-IO category of the treatment type covariate was found to be significant in univariate Cox regression. However, it is excluded from the additional multivariate analysis due to its negligible number of patients – 4.

**Table 2:** Kaplan–Meier analysis and univariate Cox regression of overall survival (OS) starting first-line of systemic treatment for patients of this study.

	Kaplan-Meier			COX		
Characteristic	3-year OS (%)	5-year OS (%)	p-value^1^	HR^2^	95% CI^2^	p-value
Gender			0.12			
Male	36.9	20.2		Ref	Ref	
Female	45.7	24.3		0.81	0.61, 1.06	0.13
Age at diagnosis			**<0.001**			
< 65	43.7	24.8		Ref	Ref	
>= 65	20.6	7.35		1.69	1.28, 2.24	**<0.001**
T stage			0.094			
T1	37.8	20.0		Ref	Ref	
T2	54.5	30.3		0.71	0.48, 1.05	0.088
T3	34.9	19.0		1.03	0.74, 1.43	0.86
T4	28.6	14.3		1.06	0.44, 2.53	0.89
N stage			0.8			
N0	40.5	23.8		Ref	Ref	
N1	29.7	18.9		1.01	0.70, 1.47	0.95
N2	38.5	23.1		1.05	0.60, 1.86	0.86
NX	39.4	17.3		1.12	0.87, 1.44	0.38
M stage			**<0.001**			
M0	44.6	25.2		Ref	Ref	
M1	14.8	4.92		2.27	1.69, 3.06	**<0.001**
MX	44.1	23.7		1.19	0.88, 1.60	0.26
Tumor Size			0.029			
0-40 mm	26.7	13.3		Ref	Ref	
40-70 mm	46.8	25.5		0.66	0.38, 1.15	0.14
70-100 mm	44.4	24.6		0.66	0.38, 1.12	0.12
> 100 mm	25.0	13.7		0.95	0.55, 1.66	0.87
Fuhrman Grade			**<0.001**			
Grade I	40.0	20.0		Ref	Ref	
Grade II	54.4	29.1		0.48	0.20, 1.19	0.11
Grade III	38.0	24.0		0.60	0.24, 1.47	0.27
Grade IV	21.8	7.69		0.90	0.37, 2.24	0.83
Microvascular Invasion			0.046			
No	46.4	23.5		Ref	Ref	
Yes	29.7	18.8		1.27	1.00, 1.60	0.047
Intra-Tumoral Necrosis			0.017			
No	48.7	25.7		Ref	Ref	
Yes	34.0	19.1		1.34	1.05, 1.70	0.017
Clear Cell Carcinoma			0.5			
No	36.4	24.2		Ref	Ref	
Yes	39.1	20.8		0.89	0.62, 1.28	0.54
Sarcomotoid Feature			**<0.001**			
No	45.4	26.1		Ref	Ref	
Yes	20.5	7.23		1.70	1.31, 2.20	**<0.001**
Treatment - 1st line			**<0.001**			
Cytotoxic Chemotherapy	53.3	26.7		Ref	Ref	
VEGF/VEGFRi* - mono	39.2	21.6		1.23	0.73, 2.07	0.44
mTORi* - mono	30.0	20.0		1.36	0.61, 3.02	0.46
IO - IO	–	–		7.84	2,56, 24.0	**<0.001**
IO - VEGF/VEGFRi*	35.0	15.0		1.50	0.77, 2.95	0.23
BMI group			0.10			
Normal	30.9	18.4		Ref	Ref	
Overweight	43.9	22.6		0.79	0.62, 1.00	0.047
Obese	48.4	25.8		0.75	0.50, 1.12	0.16
Kidney Metastases			0.2			
No	36.5	18.8		Ref	Ref	
Yes	50.0	32.1		0.82	0.61, 1.10	0.18
Lymphnodes Metastases			0.039			
No	45.2	25.4		Ref	Ref	
Yes	31.0	15.9		1.27	1.01, 1.59	0.040
Lung Metastases			0.012			
No	50.6	29.9		Ref	Ref	
Yes	34.5	17.9		1.38	1.07, 1.79	0.013
Brain Metastases			**0.002**			
No	40.1	22.1		Ref	Ref	
Yes	13.3	Ref		2.23	1.32, 3.76	**0.003**
Liver Metastases			0.11			
No	41.1	21.7		Ref	Ref	
Yes	28.8	18.6		1.27	0.95, 1.70	0.11
Bone Metastases			**0.005**			
No	41.9	23.7		Ref	Ref	
Yes	27.5	11.6		1.48	1.13, 1.94	**0.005**
Karnofsky PS			**<0.001**			
80	Ref	Ref		Ref	Ref	
90	10.5	1.75		0.23	0.13, 0.40	**<0.001**
100	48.0	27.0		0.07	0.04, 0.13	**<0.001**
Hemoglobin < LLN			**<0.001**			
No	46.6	26.1		Ref	Ref	
Yes	10.1	2.90		3.09	2.34, 4.07	**<0.001**
Serum Corr. Calcium > ULN			**<0.001**			
No	43.0	24.8		Ref	Ref	
Yes	21.9	6.25		1.90	1.44, 2.51	**<0.001**
LDH > 1.5*ULN			**<0.001**			
No	57.8	33.3		Ref	Ref	
Yes	25.1	12.3		2.02	1.60, 2.54	**<0.001**
Creatinine > ULN			0.4			
No	41.7	21.1		Ref	Ref	
Yes	29.3	21.3		1.12	0.85, 1.46	0.43
Serum Sodium < LLN			**<0.001**			
No	39.6	21.5		Ref	Ref	
Yes	Ref	Ref		3.95	1.74, 8.97	**0.001**
High PLR			0.10			
No	47.5	28.1		Ref	Ref	
Yes	32.2	15.8		1.21	0.96, 1.52	0.11
High NLR			**0.007**			
No	42.9	26.0		Ref	Ref	
Yes	32.5	13.5		1.37	1.09, 1.73	**0.007**
Number of Treatment Lines			**<0.001**			
>=3 treatment lines	55.9	31.6		Ref	Ref	
1-2 treatment lines	17.9	12.4		2.25	1.79, 2.83	**<0.001**
Toxicity, grade 3-4 - 1st line			**<0.001**			
No	33.0	15.5		Ref.	Ref.	
Yes	49.1	31.0		0.63	0.49, 0.79	**<0.001**
Dose Reduction - 1st line			**<0.001**			
No	33.9	15.6		Ref.	Ref.	
Yes	50.0	33.7		0.61	0.48, 0.78	**<0.001**
Best Response - 1st line			**<0.001**			
Progressive Disease	–	–		Ref.	Ref.	
Stable Disease	35.0	16.9		0.13	0.09, 0.20	**<0.001**
Partial Response	50.4	27.4		0.10	0.06, 0.15	**<0.001**
Complete Response	100.0	90.0		0.02	0.01, 0.06	**<0.001**

^1^Log-rank test.

^2^HR, Hazard Ratio, CI, Confidence Interval.

IO, Immuno-Oncology; i*, inhibitors.

Bold values: Significant p-value.

**Table 3** summarizes the results of the Cox multivariate model. The risk factors detected by this model with p<0.01 include M stage, brain metastases, LDH, hemoglobin, and Karnofsky performance score. Univariate analyses demonstrated a strong association between the intermediate outcomes (number of treatment lines; toxicity, dose reduction and response to the treatment in first treatment line) and survival (**Table 2**, **Supplementary Tables 1, 2**). The results of the adjusted Cox multivariate model to number of treatment lines are summarized in **Supplementary Table 3**. No major changes were demonstrated in comparison to the unadjusted model, except for the slight increase in the significance of the age of diagnosis and the bone metastases, and the slight decrease in the significance of the M stage covariate.

**Table 3:** Multivariate Cox regression of overall survival (OS) starting first-line of systemic treatment for patients of this study.

Characteristic	HR^1^	95% CI^1^	p-value
Age at diagnosis			
< 65	Ref.	Ref.	
>= 65	1.45	1.07, 1.98	0.018
M stage			
M0	Ref.	Ref.	
M1	1.63	1.15, 2.31	**0.006**
MX	0.98	0.69, 1.38	0.90
Fuhrman Grade			
Grade I	Ref.	Ref.	
Grade II	0.33	0.13, 0.84	0.020
Grade III	0.41	0.16, 1.04	0.062
Grade IV	0.19	0.05, 0.72	0.014
Sarcomotoid Feature			
No	Ref.	Ref.	
Yes	2.95	1.14, 7.60	0.025
Brain Metastases			
No	Ref.	Ref.	
Yes	2.30	1.27, 4.17	**0.006**
Bone Metastases			
No	Ref.	Ref.	
Yes	1.45	1.09, 1.94	0.011
Karnofsky PS			
80	Ref.	Ref.	
90	0.54	0.27, 1.05	0.068
100	0.26	0.13, 0.52	**<0.001**
Hemoglobin < LLN			
No	Ref.	Ref.	
Yes	1.68	1.18, 2.39	**0.004**
LDH > 1.5*ULN			
No	Ref.	Ref.	
Yes	1.53	1.18, 1.98	**0.001**

^1^HR, Hazard Ratio; CI, Confidence Interval.

Bold values: Significant p-value.

### 3.2 Model performance

**Figure 3:**
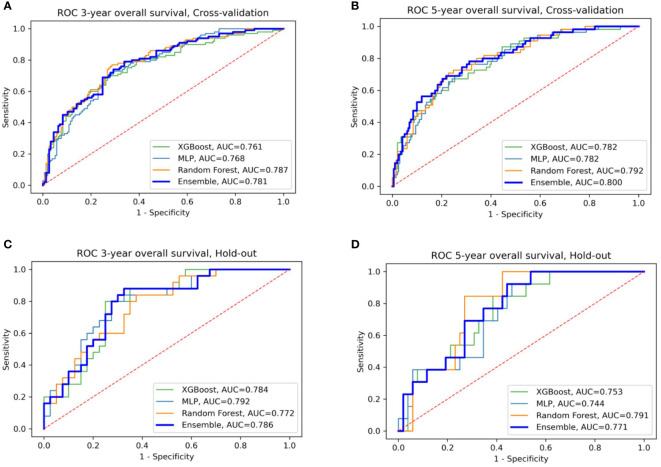
ROC curves of the four models: XGBoost, Random Forest, MLP, and their ensemble for prediction of three- and five-year OS **(A, B)** for cross-validation sets **(C, D)** for the hold-out set.

**Figure 4:**
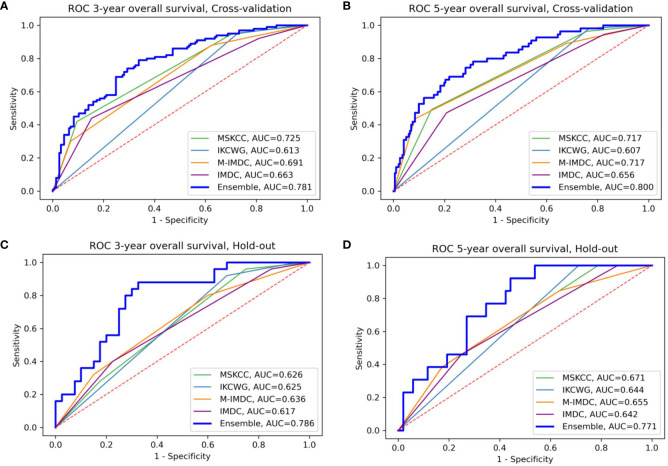
Comparison of the ensemble model and four traditional models for OS prediction for three- and five-year survival **(A, B)** for cross-validation sets **(C, D)** for the hold-out set, respectively. The area under the curve (AUC) of the ensemble model is higher than all traditional models in the four presented settings; M-IMDC stands for Modified IMDC.

We evaluated the individual models as well as the final ensemble model in five-fold cross-validation and on hold-out cohorts. Full results of the evaluation, including AUC with a 95% confidence interval and DeLong and McNemar tests, are summarized in **Table 4** for individual models, the ensemble, and four well-known prognostic models. **Figure 3** presents ROC curves of the individual and the ensemble models. **Figure 4** depicts a performance comparison between the ensemble model and the well-known prognostic models. It can be seen that the ensemble model outperforms well-known models for both outcomes in both cross-validation and hold-out sets. For example, the ensemble model for three-year survival times evaluated on the hold-out cohort obtained [AUC, 0.786 (95% CI: 0.633, 0.914)] and 0.675 specificity at sensitivity = 0.90 with significant improvement over all well-known prognostic models (DeLong p−value < 0.01, McNemar p-value < 0.01).

**Table 4:** Performance of predictive machine learning models.

Model	AUC	95% CI of AUC	DeLong test p-value	Specificity at Sensitivity = 0.90	McNemar test p-value
3-year overall survival starting first-line of systemic treatment, cross-validation					
XGBoost	0.761	0.677-0.842		0.414	
Random Forest	0.787	0.708-0.861		0.420	
MLP	0.768	0.690-0.841		0.414	
**Ensemble Model**	**0.781**	**0.702-0.856**	Ref	**0.439**	Ref
MSKCC	0.725	0.655-0.792	0.006	0.293	<0.001
IKCWG	0.613	0.574-0.651	<0.001	0.274	<0.001
IMDC	0.663	0.587-0.738	<0.001	0.191	<0.001
Modified IMDC	0.691	0.617-0.762	<0.001	0.376	0.050
3-year overall survival starting first-line of systemic treatment, hold-out					
XGBoost	0.784	0.638-0.905		0.650	
Random Forest	0.772	0.614-0.902		0.475	
MLP	0.792	0.639-0.920		0.500	
**Ensemble Model**	**0.786**	**0.633-0.914**	Ref	**0.675**	Ref
MSKCC	0.626	0.480-0.756	<0.001	0.250	<0.001
IKCWG	0.625	0.536-0.708	0.003	0.325	0.004
IMDC	0.617	0.467-0.753	0.004	0.150	0.001
Modified IMDC	0.636	0.477-0.789	0.007	0.400	0.004
5-year overall survival starting first-line of systemic treatment, cross-validation					
XGBoost	0.782	0.689-0.868		0.485	
Random Forest	0.792	0.704-0.874		0.441	
MLP	0.792	0.704-0.874		0.460	
**Ensemble Model**	**0.800**	**0.711-0.882**	Ref	**0.460**	Ref
MSKCC	0.717	0.635-0.794	0.003	0.243	<0.001
IKCWG	0.607	0.578-0.630	<0.001	0.233	<0.001
IMDC	0.656	0.568-0.744	<0.001	0.173	<0.001
Modified IMDC	0.717	0.626-0.805	<0.001	0.322	<0.001
5-year overall survival starting first-line of systemic treatment, hold-out					
XGBoost	0.753	0.582-0.899		0.481	
Random Forest	0.791	0.662-0.904		0.577	
MLP	0.744	0.579-0.885		0.538	
**Ensemble Model**	**0.771**	**0.610-0.906**	Ref	**0.558**	Ref
MSKCC	0.671	0.532-0.797	0.163	0.212	<0.001
IKCWG	0.644	0.616-0.672	0.058	0.288	0.011
IMDC	0.642	0.493-0.780	0.026	0.135	<0.001
Modified IMDC	0.655	0.465-0.828	0.024	0.365	0.007

The p-values are calculated for well-known prognostic models with respect to the ensemble model.

**Table 5** summarizes the results of subgroup analysis using the ensemble model for both outcomes. We detected several sub-populations that demonstrate better discrimination performance of the model than in the whole cross-validation set. These groups outperformed the overall AUC reported on cross-validation cohort by at least 5 points and had sizes of not less than 50 patients. Validation on these sub-populations in the hold-out set didn’t bring reliable results due to the small size of this cohort.

**Table 5:** Subgroup analysis - performance of the ensemble model in selected subgroups for three- and five-year overall survival.

Sub-group	# Patients in sub-group	AUC	95% CI of AUC
3-year overall survival starting first-line of systemic treatment			
Females	56	0.866	0.742-0.938
No lung metastases	73	0.849	0.742-0.919
Bone metastases	51	0.876	0.747-0.947
5-year overall survival starting first-line of systemic treatment			
T stage is pT1 or pT2	84	0.852	0.754-0.917
Metastases in lymph nodes	119	0.879	0.803-0.929
Number of metastatic sites > 2	179	0.845	0.782-0.893
Hemoglobin < LLN	54	0.961	0.860-0.993

**Figure 5:**
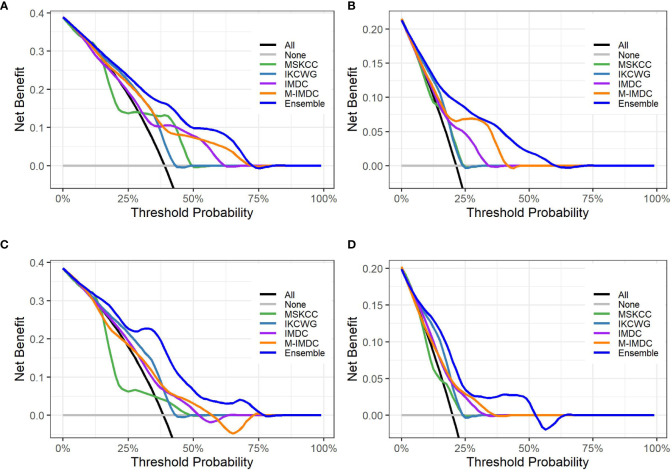
Decision curve analysis graphs showing the net benefit produced by the models across all threshold probabilities. The depicted curves were obtained using predictions of the ensemble model compared with well-known prognostic models, as well as two additional curves that were based on two types of extreme decisions: curves referred to as ‘All’ represent the prediction that all patients would die in three to five years after treatment start. The curve referred to as ‘None’ represents the prediction that all the patients would be alive in three to five years after treatment starts. **(A, B)** show the decision curves of predictive models for the cross-validation set for three- and five-year OS, respectively. **(C, D)** show the decision curves of predictive models for the hold-out set for three- and five-year OS, respectively. The decision curve indicates that the ensemble model has a higher benefit than the prediction that all patients will die, or none will die; it is higher than all well-known models for the reasonable range of thresholds.

**Figure 5** presents graphs of DCA for the ensemble model compared with four well-known prognostic models for three- and five-year overall survival. The y-axis of the decision curve represents the net benefit, which is used to decide whether any specific clinical decision result gives more benefit than harm. The x-axis represents threshold probabilities that differentiate between dead and live patients. It can be seen that the ensemble model has a higher net clinical benefit than the traditional models for cross-validation and hold-out sets for both the three-year and five-year survival outcomes.

In addition, we demonstrated the validity of the developed ensemble model in both sub-cohorts with small (1-2) and large (more than 3) number of treatment lines by obtaining an AUC of above 0.7 in cross-validation and hold-out data sets for both outcomes. Detailed results can be found in **Supplementary Table 4**.

### 3.3 Model explainability with SHAP analysis

**Figure 6:**
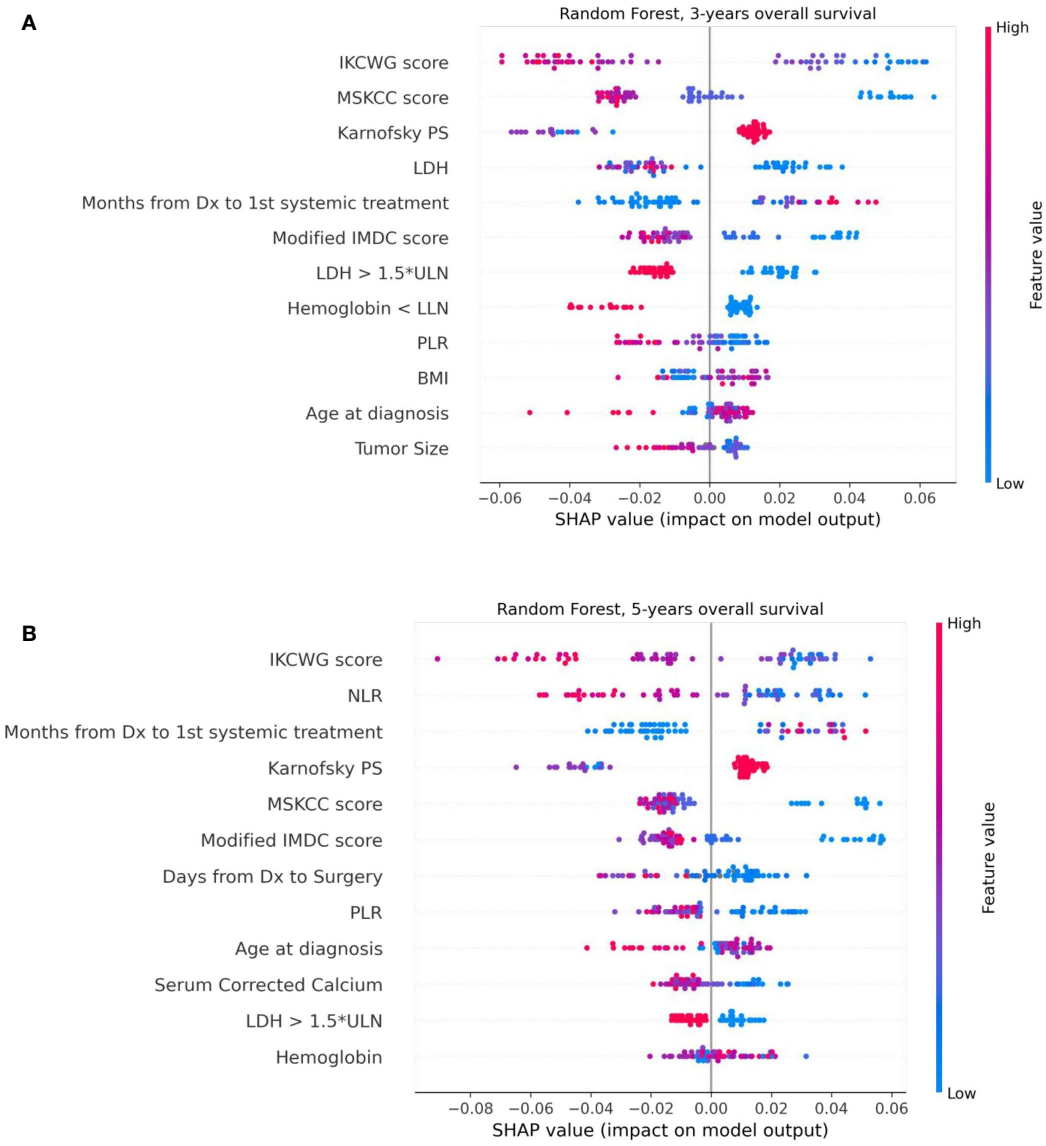
The Shapley additive explanations summary plots of 12 top features of the Random Forest model, ordered by their impact on 3-year **(A)** and 5-year **(B)** survival, respectively.

We used the SHAP method for explainability of the predictive machine learning models. The SHAP summary plot presents features ordered top-down based on their impact on the outcome (three- and five-year survival). The SHAP values of a feature are correlated to the possibility of survival: positive SHAP value means positive impact on the prediction, while negative ones lead the model to predict “no survival”. The dot color represents the values that each feature can take: red for high values, blue for low values, and purple for values that are close to the average value. **Figure 6** shows the SHAP explainability for Random Forest for survival outcomes. Consider the “Karnofsky PS’’ feature from **Figure 6A** as an example. We see that this feature is important for the model since it appears in the third position from the top. The higher (red) values of this feature are associated with a higher likelihood of survival. Patients with lower (blue) and average (purple) feature values have stronger influence on the negative outcome. We see that the scores of well-known prognostic models, such as MSKCC, Modified IMDC, and IKCWG are important discriminators in the machine learning models as they appear at the top of the plots. This can be explained by accumulated knowledge encoded in their scoring mechanism that was evaluated on a large number of patients. In addition, the time between diagnosis to systemic treatment appears to be important for both outcomes. SHAP demonstrates that its high and average values correlate with survival, while low values do not. This can be explained as follows. Low values (blue points) of time from diagnosis to systemic treatment are associated with a synchronous metastatic disease, i.e. (usually) a more aggressive disease. High (red) values relate to patients for whom mRCC was diagnosed metachronously during the follow-up, possibly suggesting (at least on average) a more indolent disease.

## 4 Discussion

In this retrospective analysis, we developed machine-learning-based predictive models that incorporate clinical factors for the individualized prediction of three- and five-year survival for mRCC patients starting a first-line of systemic treatment. To the best of our knowledge, these are the first AI-based prediction models that were developed for this task. The proposed models show favorable discrimination in cross-validation (AUC 0.781 and 0.800 for three- and five-year survival, respectively) and hold-out cohorts (AUC 0.786 and 0.771 for three- and five-year survival, respectively), with the demonstration of better predictive performance than well-known prognostic models for both cohorts and outcomes. For example, the proposed model for three-year survival outperforms the best of the well-known models (Modified IMDC) on the same cohort; it has an outcome with the AUC metric (0.786 vs 0.636, DeLong test p-value = 0.007) and with specificity for sensitivity=90 (0.675 vs 0.40, McNemar test p-value = 0.004). The presentation of good specificity results in high sensitivity operations is a good criteria for the model’s deployment in clinical practice. Our further analysis of model performance in the cross-validation cohort revealed that for several specific sub-populations, our models demonstrated even better discrimination than on the whole cross-validation cohort. For example, low T stages, patients with metastases in lymph nodes, large number of metastatic sites, and low hemoglobin gives better discrimination for the five-year survival model. For the three-year survival model, this validation achieved high performance for women patients without lung metastases or with bone metastases. These results should be regarded as hypotheses-generating and would require both further investigations, as well as validation using larger cohorts in future work. The decision curve analysis revealed that the proposed model had a higher overall net benefit than four well-known prognostic models in predicting both three-year and five-year survival for the ranges of reasonable threshold probabilities in both cross-validation and hold-out cohorts. The highest clinical net benefit, meaning the highest value of benefits minus drawbacks, is clearly significant. Therefore, using this model in clinical practice may reduce the cases of overtreatments and the number of redundant follow-ups. The SHAP analysis that was performed for the model’s explainability demonstrated no significant difference in feature importance between models for both outcomes. This analysis revealed that models for both outcomes significantly benefited from the enriched features of risk scores of well-known models. We can see that the scores of the IKCWG, MSKCC, and Modified IMDC models appear among the top features. In addition, for both models, LDH, PLR, Karnofsky patient performance, hemoglobin and time from diagnosis to first-line treatment appear to be significant. Three of these features, LDH, Karnofsky patient performance and hemoglobin, demonstrated significance in all of the applied methods of statistical and survival analyses. Significance of time from diagnosis to first systemic treatment was only missing in multivariate Cox models; the PLR was significant only in univariate analysis for both outcomes. (**Tables 2**, **3**, **Supplementary Tables 1-3**).

The validity of the developed model is also supported by the well-known prognostic models which include the same top risk factors as our model. Recent works on risk factors in RCC survival (38, 39) indicate potential new features, like time to recurrence and renal function, wich can be investigated in future studies.

As a whole, this study suggests that a machine-learning-based model can better predict the outcome (in terms of three- and five-year survival) of mRCC patients, as compared to standard prognostication systems in a real-world setting. Augmenting the care process with these AI models can lead to improved patient care and management. Clearly, the advantage of this model should be weighed against its practical feasibility, to take into account several limitations of our case series. Indeed, this is a single-institution retrospective series that included patients treated mainly with monotherapy. These treatments were mainly represented by “old” targeted agents, while the present standard of treatment is represented by immune-based combinations. Thus, a prospective validation on a larger, multicentered, series of mRCC treated with the present standards of treatment would be warranted.

This experience opens the need for further refinements. It is intriguing to imagine the potential of adding to the present amount of clinical data – just a touch of more complex tumor biology with more in-depth features such as genomic alterations or signatures to predict the suitability of immune-based therapies, PD-L1 expression, mutational burden, and other.

We cannot propose to substitute presently available prognostication models with this AI-based model, but it crystal clear in our mind that this will be the future, when we will be able to routinely combine different levels of biological insights of the tumors affecting our patients.

## Data availability statement

The data analyzed in this study is subject to the following licenses/restrictions: This data set is a private data set belonging to the Italian hospitals involved in the CAPABLE project (European Union’s Horizon 2020 research and innovation programme under grant agreement No 875052). Requests to access these datasets should be directed to valentina.tibollo@icsmaugeri.it.

## Ethics statement

The studies involving human participants were reviewed and approved by the Ethics Committee of IRCCS Istituti Clinici Scientifici Maugeri (Approval Number: 2421 CE, on February 23rd, 2020). Written informed consent for participation was not required for this study in accordance with the national legislation and the institutional requirements.

## Author contributions

CP, MR, EB, SQ: Conceived and designed the study. EB, CP, MR, SQ, SR-C: Drafted the manuscript. MR, VT: Collected, assembled, interpreted the data EB, SR-C: Processed and analyzed the data All authors contributed to the article and approved the submitted version.
