# Supplementary material for: Artificial intelligence-based prediction of overall survival in metastatic renal cell carcinoma
Source: Front Oncol. 2023 Feb 16;13:1021684. doi: 10.3389/fonc.2023.1021684 (PMC9978529; doi:10.3389/fonc.2023.1021684)
Supplement: Supplementary file 1 [file DataSheet_1.pdf]

Supplementary Material

Supplementary Table 1: Univariate analysis for 3-year overall survival (OS) starting first-line of systemic treatment for this study of patients.

**Characteristic**	**Survivors, N = 125[1]**	**Non-Survivors, N = 197[1]**	**p-value[2]**
Months from Dx to 1st line	31.9 (7.9, 86.1)	9.4 (3.2, 29.4)	<0.001
Days from Dx to Surgery	17.0 (8.0, 28.0)	21.0 (10.2, 36.0)	0.067
Treatment - 1st line			0.42
VEGF/VEGFRi* - mono	107 (86%)	166 (84%)	
mTORi* - mono	3 (2.4%)	7 (3.6%)	
IO - IO	0 (0%)	4 (2.0%)	
IO - VEGF/VEGFRi*	7 (5.6%)	13 (6.6%)	
Cytotoxic Chemotherapy	8 (6.4%)	7 (3.6%)	
Gender			0.18
Male	93 (74%)	159 (81%)	
Female	32 (26%)	38 (19%)	
Age at diagnosis			<0.001
< 65	111 (89%)	143 (73%)	
>= 65	14 (11%)	54 (27%)	
BMI group			0.039
Normal	42 (34%)	94 (48%)	
Overweight	68 (54%)	87 (44%)	
Obese	15 (12%)	16 (8.1%)	
Karnofsky PS			<0.001
80	0 (0%)	17 (8.6%)	
90	6 (4.8%)	51 (26%)	
100	119 (95%)	129 (65%)	
T stage			0.038
T1	17 (14%)	28 (15%)	
T2	36 (30%)	30 (16%)	
T3	66 (55%)	123 (66%)	
T4	2 (1.7%)	5 (2.7%)	
M stage at diagnosis			<0.001
M0	90 (72%)	112 (57%)	
M1	9 (7.2%)	52 (26%)	
MX	26 (21%)	33 (17%)	
N stage			0.68
N0	68 (54%)	100 (51%)	
N1	11 (8.8%)	26 (13%)	
N2	5 (4.0%)	8 (4.1%)	
NX	41 (33%)	63 (32%)	
Tumor Size			0.009
0-40 mm	4 (3.2%)	11 (5.8%)	
40-70 mm	44 (35%)	50 (26%)	
70-100 mm	56 (45%)	70 (37%)	
> 100 mm	20 (16%)	60 (31%)	
Fuhrman Grade			<0.001

**Supplementary Table 1: Univariate analysis for 3-year overall survival (OS) starting first-line of systemic treatment for this study of patients.**			
**Characteristic**	**Survivors, N = 125[1]**	**Non-Survivors, N = 197[1]**	**p-value[2]**
Grade I	2 (1.7%)	3 (1.6%)	
Grade II	56 (46%)	47 (25%)	
Grade III	46 (38%)	75 (40%)	
Grade IV	17 (14%)	61 (33%)	
Microvascular Invasion	38 (31%)	90 (48%)	0.003
Intra-Tumoral Necrosis	66 (55%)	128 (69%)	0.011
Clear Cell Carcinoma	113 (90%)	176 (89%)	0.76
Sarcomotoid Feature	17 (14%)	66 (34%)	<0.001
Kidney Metastases	28 (22%)	28 (14%)	0.059
Lymphnodes Metastases	45 (36%)	100 (51%)	0.009
Lung Metastases	81 (65%)	154 (78%)	0.008
Brain Metastases	2 (1.6%)	13 (6.6%)	0.038
Liver Metastases	17 (14%)	42 (21%)	0.081
Bone Metastases	19 (15%)	50 (25%)	0.030
Hemoglobin g/dl	14.1 (13.1, 14.9)	13.5 (12.5, 14.4)	<0.001
Serum Corr. Calcium mg/dl	9.7 (9.2, 10.1)	9.8 (9.4, 10.3)	0.011
LDH mU/ml	210.0 (188.0, 250.0)	256.0 (212.0, 362.0)	<0.001
PLR	148.1 (120.6, 205.0)	178.5 (126.5, 233.3)	0.006
NLR	3.3 (2.7, 4.0)	3.5 (3.0, 4.4)	0.010
Serum Sodium(Na) mmol/l	141.0 (139.0, 144.0)	141.0 (139.0, 144.0)	0.10
Creatinine	1.1 (0.9, 1.3)	1.1 (1.0, 1.3)	0.036
Best Response - 1st line			<0.001
Progressive Disease	0 (0%)	35 (18%)	
Stable Disease	56 (45%)	104 (53%)	
Partial Response	59 (47%)	58 (29%)	
Complete Response	10 (8.0%)	0 (0%)	
Toxicity, grade 3-4 - 1st line	57 (46%)	59 (30%)	0.004
Dose Reduction - 1st line	49 (39%)	49 (25%)	0.006
Number of Treatment Lines	3.0 (3.0, 4.0)	2.0 (2.0, 3.0)	<0.001

[1]Median (IQR); n (%)

[2]Wilcoxon rank sum test; Pearson's Chi-squared test; Fisher's exact test

Abbreviations: IO – Immuno-Oncology, i* - inhibitors

Supplementary Table 2: Univariate analysis for 5-year overall survival (OS) starting first-line of systemic treatment for this study of patients.

Characteristic	Survivors, N = 68[1]	Non-Survivors, N = 254[1]	p-value[2]
Months from Dx to 1st line	35.9 (11.7, 88.2)	10.3 (3.5, 38.6)	<0.001
Days from Dx to Surgery	16.0 (8.2, 24.8)	20.0 (10.0, 35.0)	0.094
Treatment - 1st line			0.86
VEGF/VEGFRi* - mono	59 (87%)	214 (84%)	
mTORi* - mono	2 (2.9%)	8 (3.1%)	
IO - IO	0 (0%)	4 (1.6%)	
IO - VEGF/VEGFRi*	3 (4.4%)	17 (6.7%)	
Cytotoxic Chemotherapy	4 (5.9%)	11 (4.3%)	
Gender			0.46
Male	51 (75%)	201 (79%)	
Female	17 (25%)	53 (21%)	
Age at diagnosis			0.002
< 65	63 (93%)	191 (75%)	
>= 65	5 (7.4%)	63 (25%)	
BMI group			0.54
Normal	25 (37%)	111 (44%)	
Overweight	35 (51%)	120 (47%)	
Obese	8 (12%)	23 (9.1%)	
Karnofsky PS			<0.001
80	0 (0%)	17 (6.7%)	
90	1 (1.5%)	56 (22%)	
100	67 (99%)	181 (71%)	
T stage			0.27
T1	9 (14%)	36 (15%)	
T2	20 (30%)	46 (19%)	
T3	36 (55%)	153 (63%)	
T4	1 (1.5%)	6 (2.5%)	
M stage at diagnosis			0.003
M0	51 (75%)	151 (59%)	
M1	3 (4.4%)	58 (23%)	
MX	14 (21%)	45 (18%)	
N stage			0.60
N0	40 (59%)	128 (50%)	
N1	7 (10%)	30 (12%)	
N2	3 (4.4%)	10 (3.9%)	
NX	18 (26%)	86 (34%)	
Tumor Size			0.18
0-40 mm	2 (2.9%)	13 (5.3%)	
40-70 mm	24 (35%)	70 (28%)	
70-100 mm	31 (46%)	95 (38%)	
> 100 mm	11 (16%)	69 (28%)	
Fuhrman Grade			0.002

Supplementary Table 2: Univariate analysis for 5-year overall survival (OS) starting first-line of systemic treatment for this study of patients.			
**Characteristic**	**Survivors, N = 68[1]**	**Non-Survivors, N = 254[1]**	**p-value[2]**
Grade I	1 (1.5%)	4 (1.7%)	
Grade II	30 (45%)	73 (30%)	
Grade III	29 (44%)	92 (38%)	
Grade IV	6 (9.1%)	72 (30%)	
Microvascular Invasion	24 (36%)	104 (43%)	0.32
Intra-Tumoral Necrosis	37 (56%)	157 (65%)	0.18
Clear Cell Carcinoma	60 (88%)	229 (90%)	0.64
Sarcomotoid Feature	6 (8.8%)	77 (30%)	<0.001
Kidney Metastases	18 (26%)	38 (15%)	0.026
Lymphnodes Metastases	23 (34%)	122 (48%)	0.036
Lung Metastases	42 (62%)	193 (76%)	0.019
Brain Metastases	0 (0%)	15 (5.9%)	0.047
Liver Metastases	11 (16%)	48 (19%)	0.61
Bone Metastases	8 (12%)	61 (24%)	0.029
Hemoglobin g/dl	14.2 (13.3, 15.0)	13.7 (12.5, 14.5)	0.001
Serum Corr. Calcium mg/dl	9.7 (9.2, 10.1)	9.8 (9.4, 10.2)	0.024
LDH mU/ml	206.5 (185.8, 240.2)	242.5 (204.0, 337.0)	<0.001
PLR	139.5 (109.2, 198.8)	175.4 (128.0, 223.2)	0.003
NLR	3.2 (2.5, 3.6)	3.5 (3.0, 4.4)	<0.001
Serum Sodium (Na) mmol/l	141.0 (140.0, 144.0)	141.0 (139.0, 144.0)	0.37
Creatinine	1.2 (1.0, 1.3)	1.1 (0.9, 1.3)	0.86
Best Response - 1st line			<0.001
Progressive Disease	0 (0%)	35 (14%)	
Stable Disease	27 (40%)	133 (52%)	
Partial Response	32 (47%)	85 (33%)	
Complete Response	9 (13%)	1 (0.4%)	
Toxicity, grade 3-4 - 1st line	36 (53%)	80 (31%)	0.001
Dose Reduction - 1st line	33 (49%)	65 (26%)	<0.001
Number of Treatment Lines	3.0 (3.0, 4.0)	2.0 (2.0, 3.0)	<0.001

[1]Median (IQR); n (%)

[2]Wilcoxon rank sum test; Pearson's Chi-squared test; Fisher's exact test

Abbreviations: IO – Immuno-Oncology, i* - inhibitors

Supplementary Table 3. Multivariate Cox regression of overall survival (OS) starting first-line of systemic treatment adjusted to the number of treatment lines.
Bold values: Significant p-value.

Characteristic	HR[1]	95% CI[1]	p-value
Age at diagnosis			
< 65	Ref.	Ref.	
>= 65	1.69	1.25, 2.31	**<0.001**
M stage			
M0	Ref.	Ref.	
M1	1.55	1.07, 2.24	0.020
MX	0.96	0.68, 1.36	0.82
Fuhrman Grade			
Grade I	Ref.	Ref.	
Grade II	0.31	0.12, 0.80	0.015
Grade III	0.37	0.15, 0.95	0.038
Grade IV	0.26	0.07, 1.00	0.050
Sarcomotoid Feature			
No	Ref.	Ref.	
Yes	2.82	1.05, 7.55	0.039
Brain Metastases			
No	Ref.	Ref.	
Yes	2.67	1.44, 4.93	**0.002**
Bone Metastases			
No	Ref.	Ref.	
Yes	1.91	1.42, 2.57	**<0.001**
Karnofsky PS			
80	Ref.	Ref.	
90	0.71	0.35, 1.42	0.33
100	0.22	0.11, 0.45	**<0.001**
Hemoglobin < LLN			
No	Ref.	Ref.	
Yes	1.79	1.25, 2.54	**0.001**
LDH > 1.5*ULN			
No	Ref.	Ref.	
Yes	1.45	1.12, 1.90	**0.006**

[1]HR = Hazard Ratio, CI = Confidence Interval

Supplementary Table 4. Performance of the ensemble model in two sub-cohorts corresponding to the number of treatment lines for three- and five-year overall survival in cross-validation and hold-out data sets

	Low number of lines (1-2)			High number of lines (>=3)		
**Sub-Cohort**	**#Patients in sub-group**	**AUC**	**95% CI of AUC**	**#Patients in sub-group**	**AUC**	**95% CI of AUC**
3-year survival – cross-validation data set	118	0.814	0.730-0.877	139	0.739	0.656-0.807
3-year survival – hold-out data set	27	0.717	0.510-0.865	38	0.782	0.614-0.893
5-year survival – hold-out data set	118	0.805	0.720-0.870	139	0.773	0.693-0.838
5-year survival – hold-out data set	27	0.962	0.789-0.998	38	0.715	0.543-0.843
